# A Case of Anti-glomerular Basement Disease Without Pulmonary Involvement

**DOI:** 10.7759/cureus.4130

**Published:** 2019-02-25

**Authors:** Adeel Nasrullah, Zainab Fatima, Anam Javed, Usman Tariq, Muhammad Sabih Saleem

**Affiliations:** 1 Internal Medicine, Allegheny General Hospital, Pittsburgh, USA; 2 Internal Medicine, Shifa International Hospital, Islamabad, PAK; 3 Internal Medicine, University College of Medicine and Dentistry, Lahore, PAK; 4 Internal Medicine, Yale University School of Medicine, New Haven, USA

**Keywords:** goodpasture`s syndrome, glomerulo nephritis, autoimmune disease, glomerular basement membrane, anti-glomerular basement disease

## Abstract

Anti-glomerular basement membrane disease is a rare but classic example of an antibody-mediated disease. The scale of injury that it entails depends on the site where the antibodies are deposited, with some patients presenting with a composite of pulmonary and renal damage. In other scenarios, the renal system is the main site of affliction with patients deteriorating to a status of acute renal failure within days of diagnosis. Due to the paucity of its incidence, we present our findings of anti-glomerular basement disease with pulmonary sparing. Herein, we also review the array of different physical findings, different forms of perpetrating antibodies, the diagnostic tools at our disposal, and the treatment modalities utilized to prevent catastrophic tissue injuries.

## Introduction

Anti-glomerular basement membrane disease is a rare autoimmune illness that develops secondary to the deposition of autoantibodies that line the basement membranes of the renal glomeruli, leading to rapidly progressive glomerulonephritis. The incidence of this disease stands at one case per one million per annum, which underlines the rarity of this ailment [1]. The circulating autoantibodies may also line the alveolar basement membranes and instigate pulmonary injury, which can present with varying degrees of pulmonary hemorrhage. This combination of findings is more specifically known as Goodpasture’s syndrome [2]. Herein, we present a case of anti-glomerular basement membrane disease without pulmonary involvement and a discussion on the management of our patient as well as the different treatment modalities used to curb the affiliated kidney injury. 

## Case presentation

An 86-year-old male with a decade-old history of prostate cancer that mitigated with subsequent radiation therapy presented to a local hospital with complaints of black stools for one day as well as an episode of syncope. Further inquiry revealed that his current predicament had been preceded by bouts of nausea and poor appetite for the last four weeks, supplemented with an unintentional weight loss of fifteen pounds in the previous six months.

**Figure 1:**
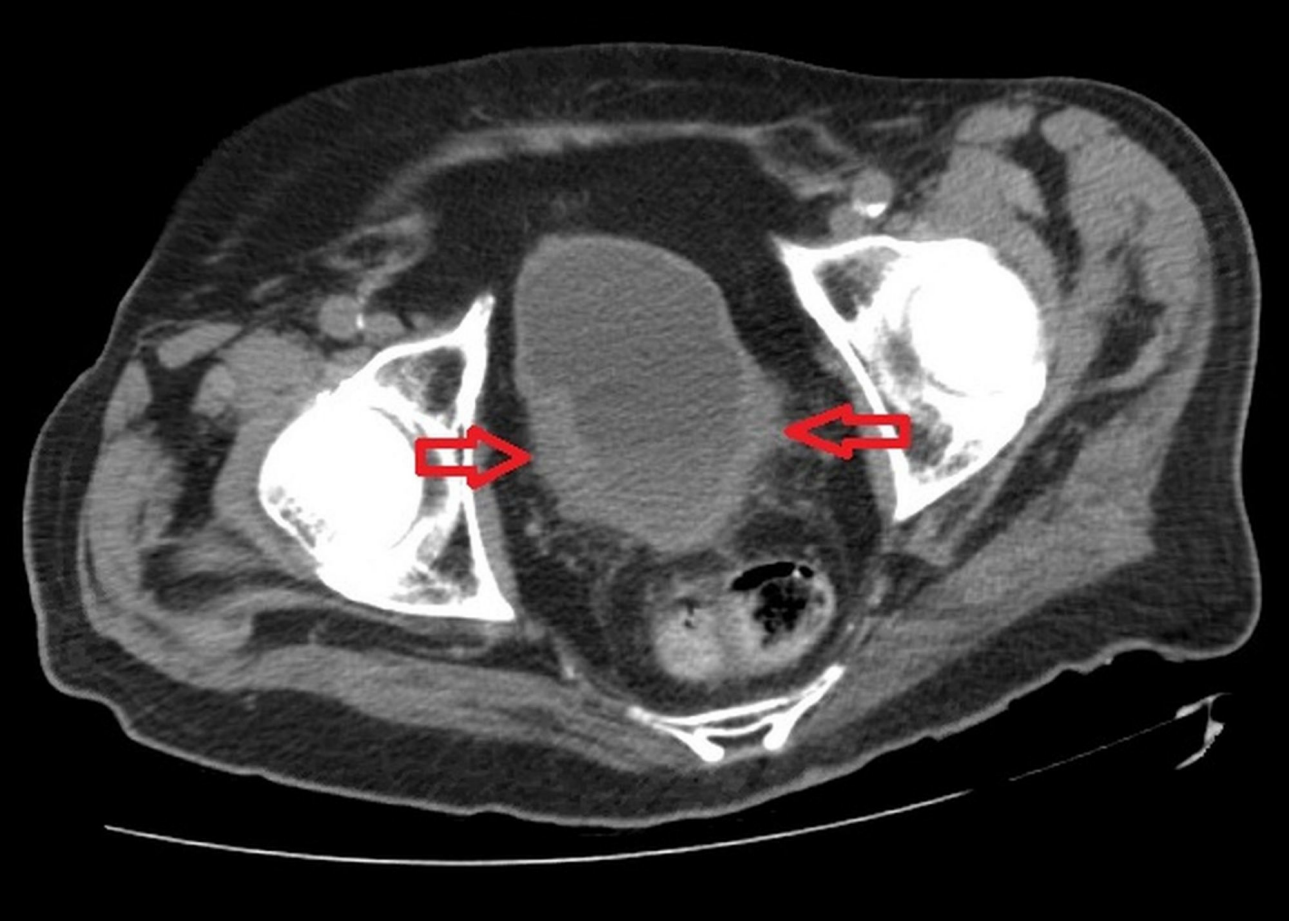
A non-contrast computerized tomography scan of the abdomen and pelvis showing an asymmetrical thickening of the bladder wall (red arrows).

Initial laboratory workup revealed a low hemoglobin of 11 mg/dL, a considerably elevated serum creatinine of 14.83 mg/dL (from a baseline of 1.00 mg/dL), while urinalysis (UA) eluded to a large amount of dysmorphic red blood cells (RBCs), 2+ urine protein but no evidence of an underlying urinary tract infection (UTI). A resultant renal ultrasound ruled out obstructive uropathy as the perpetrating cause. The patient underwent two separate sessions of hemodialysis which reduced the serum creatinine to baseline levels. A prior history of prostate cancer warranted the use of a non-contrast computerized tomography (CT) scan of the abdomen and pelvis which revealed new osteoblastic lesions in the L2 vertebra as well as an asymmetrical thickening of the bladder wall which was concerning for a recurred metastatic disease (Figure 1).

**Figure 2:**
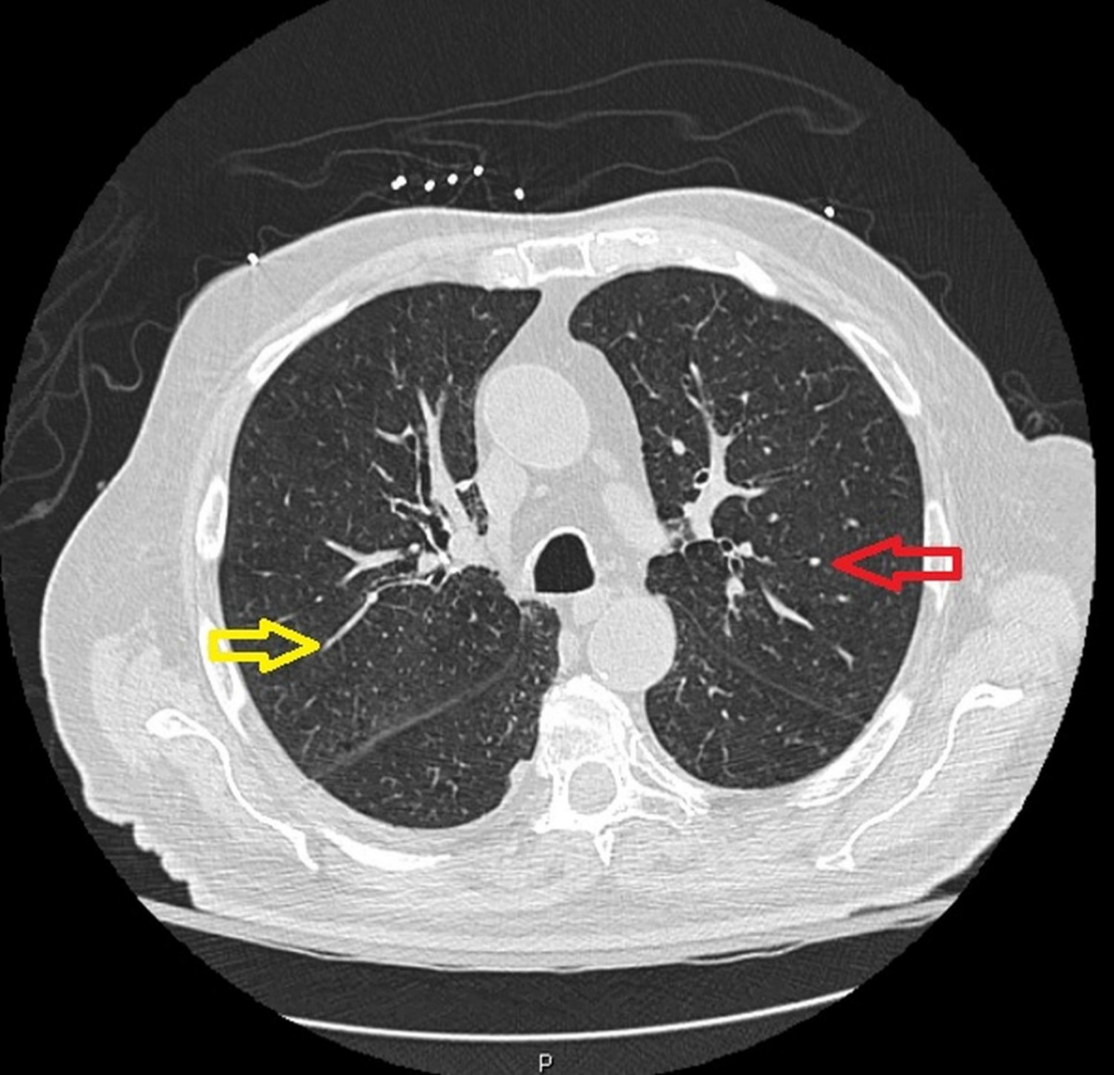
Non-contrast computed tomography scan of the chest showing bilateral pulmonary nodules (red arrow) and interstitial infiltrates (yellow arrow).

During the course of his admission, the patient frequently passed melanotic stools which precipitated to a second syncopal episode with a drastic decline in hemoglobin levels to 6 mg/dL. The patient was subsequently transferred to our medical facility for further management. He was initially transfused with two units of packed RBCs and then later underwent an esophagogastroduodenoscopy (EGD) which revealed a non-bleeding duodenal ulcer that was remedied with bipolar cautery and clipping. Following the EGD, the patient was noted to have shortness of breath (SOB). A subsequent chest CT scan without contrast revealed emphysematous changes in bilateral lung fields, with interstitial fibrosis and nodular formations (Figure 2). 

Owing to the inference of an underlying autoimmune etiology, the patient underwent an autoimmune workup (Table 1). 

**Table 1:** Autoimmune profile P-ANCA: perinuclear anti-neutrophil cytoplasmic antibodies; C-ANCA: cytoplasmic antineutrophil cytoplasmic antibodies; MPO: myeloperoxidase, PR3: proteinase 3; GBM: glomerular basement membrane, C3: compliment 3; C4: compliment 4; ANA: anti-nuclear antibody; Anti-dsDNA: anti-double stranded deoxyribonucleic acid.

Antibody	Normal Value	Result
P-ANCA	< 1:20	1:640
C-ANCA	< 1:20	1:80
MPO antibodies	0-9 IU/mL	<9 IU/mL
PR3 antibodies	0-3.5 IU/mL	<3.5 IU/mL
Anti-GBM antibodies	0-20 U/mL	182 U/mL
C3	85-193 mg/dL	110.9 mg/dL
C4	12-36 mg/dL	21.8 mg/dL
ANA	<1:80	1:320
Anti-dsDNA antibody	-	Negative

**Figure 3:**
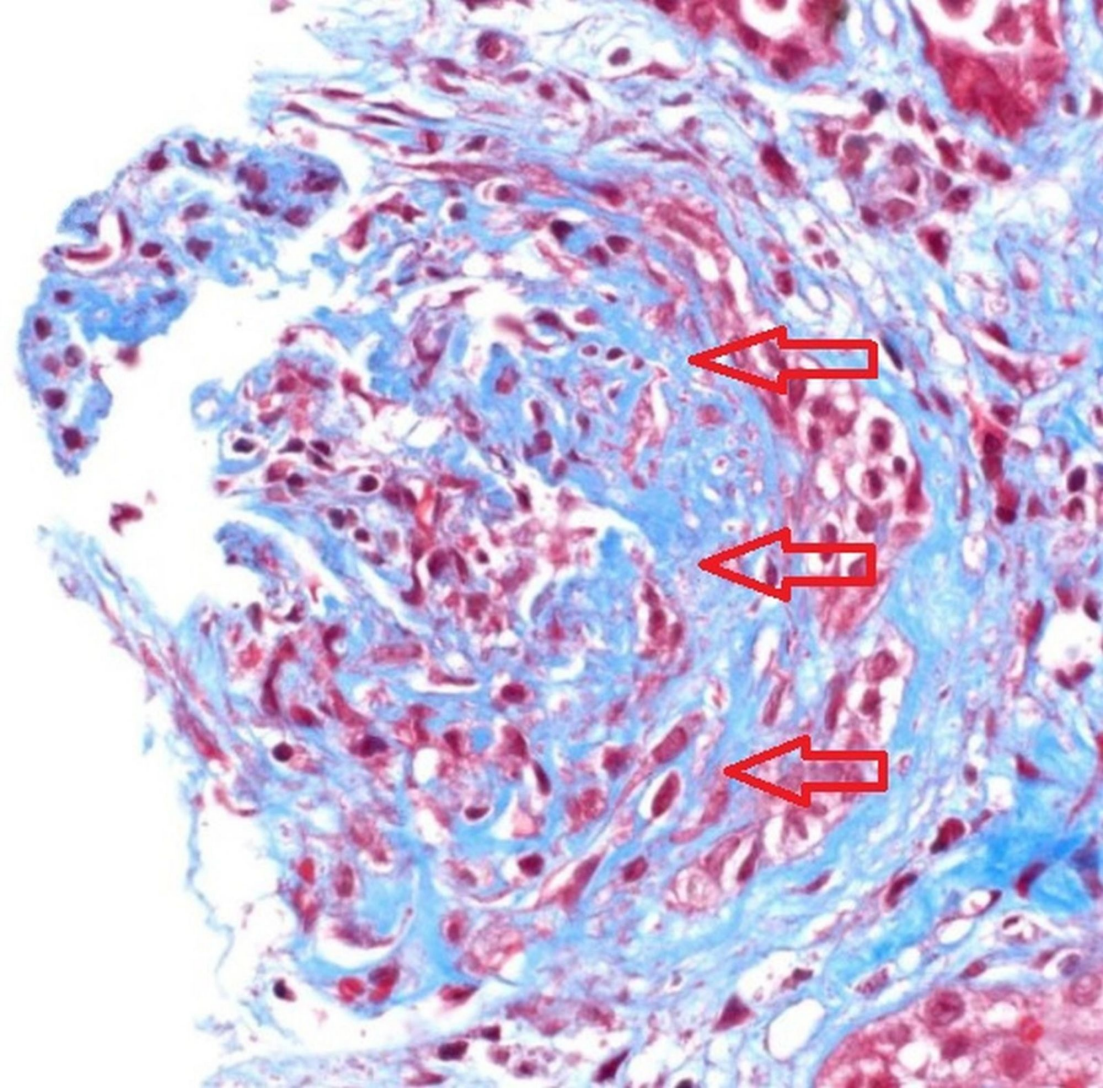
A hematoxylin-eosin stained renal biopsy specimen revealing a crescent formation characterized by cellular necrosis, inflammatory infiltrate in the interstitium, and sloughing of the tubular epithelium (red arrows).

**Figure 4:**
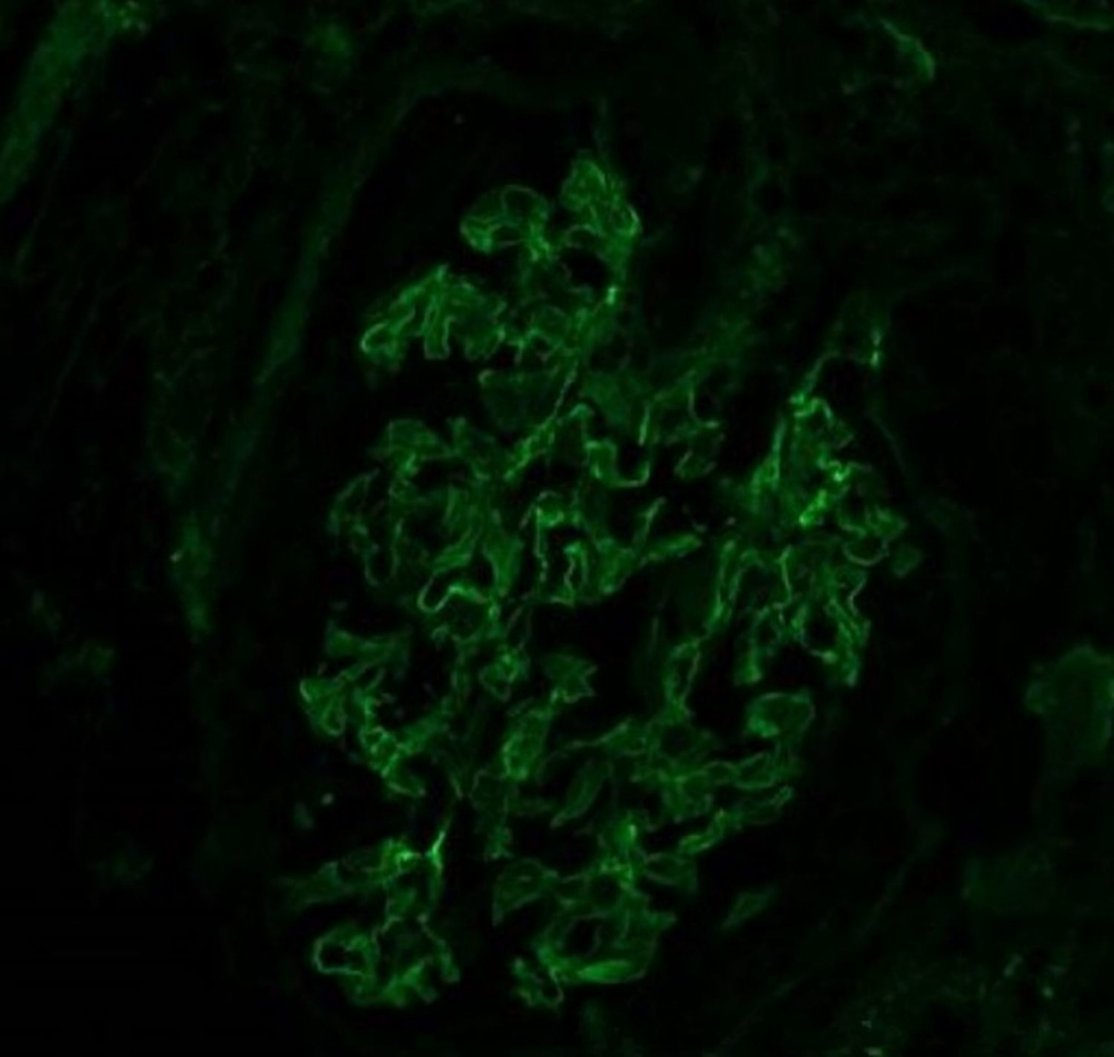
Immunofluorescence of the histological section showed linear deposits along the basement membrane.

A high titer of autoantibodies in the autoimmune profile further suggested that the underlying cause of the patient's findings can be attributed to a pathology of anti-glomerular basement membrane disease which was later confirmed via a left renal biopsy which showed necrotizing granulomatous crescentic glomerulonephritis and an immunofluorescence of the histological section which showed linear deposits along the basement membrane (Figures 3-4).

We subsequently established a diagnosis of anti-glomerular basement membrane disease. The patient was managed on the same line with an initial administration of steroid pulse therapy with methylprednisolone for three days followed by an oral administration of 60 mg prednisone for one week. He was also started on a renal-adjusted course of vancomycin and ciprofloxacin because of elevated levels of procalcitonin and scheduled for five sessions of plasmapheresis with concomitant hemodialysis owing to his declining renal function which precipitated to considerable lethargy. Following a harm-benefit analysis, the fifth session of plasmapheresis was abandoned. Prednisone was tapered to 30 mg on the fourteenth day of admission with an aim to tail off the dosage by 10 mg every week.

A tunneled catheter was placed for subsequent outpatient dialysis visits and the patient was discharged to hospice after fourteen days of inpatient care.

## Discussion

Anti-glomerular basement membrane (anti-GBM) disease is a classic auto-antibody mediated small vessel vasculitis. The circulating antibodies involved in anti-GBM disease are almost exclusively IgG and they are mainly directed against the alpha three non-collagenous domain (NC1) of type-four collagen [3].

Although anti-alpha three antibodies are exclusively present in all patients, some patients also have antibodies directed against other collagen chains such as alpha four and alpha five. The presence of circulating antibodies is further supported by the improvement in the clinical outcome of these patients following plasmapheresis. These circulating antibodies can potentially damage any renal allograft, explaining the reason behind unsuccessful kidney transplants in patients with the anti-GBM disease. This implicates the antibodies in the pathogenesis of the disease process and shows that they are not merely markers for the ongoing pathology. Recent studies on animal models have also highlighted the role of T-lymphocytes in the disease pathogenesis. These studies suggest that T-cells can cause direct cell-mediated glomerular injury and therefore T-lymphocytes may be present in kidney biopsy samples [4]. 

Anti-GBM disease is a rare entity with an incidence of 0.1 cases per million. The disease is more common in Caucasians than in African-Americans and exhibits a bimodal age distribution with the first peak at age 30 years and the second one at 60 years of age. Most cases of anti-GBM disease are associated with either an insult to the basement membrane or cross-reactivity with exogenous epitopes. Some of the events including infections, toxins, neoplasia, renal injury, and endogenous antigens can also trigger anti-GBM disease in genetically susceptible individuals [5]. While the instigating factors for the illness can normally be determined, we could not elucidate the initiating cause for our patient's renal pathology. 

The clinical spectrum ranges from mild renal involvement to rapidly progressive glomerulonephritis with findings of acute renal failure. In about 40-60% of the patients, the anti-GBM disease also involves the lungs, with patients usually presenting with varying manifestations that can range from overt pulmonary hemorrhaging to inconspicuous bleeding. This combination of glomerulonephritis and pulmonary hemorrhage is referred to as Goodpasture syndrome. Isolated renal involvement is more uniquely observed in patients over fifty years of age, who present with a less indolent course [4,6].

Drastically elevated serum creatinine supplemented with dysmorphic RBCs and profound proteinuria on urinalysis indicated significant renal damage in our patient which was consistent with a diagnosis of rapidly progressive glomerulonephritis. Unlike renal involvement, symptoms of pulmonary involvement are non-specific and can range from blood streaked hemoptysis to massive pulmonary hemorrhage [5]. GBM antibodies are detected using direct enzyme-linked immunoassay (ELISA) and further confirmed via a western blot for confirmation. False negative results can occur in patients with a low antibody titer or those afflicted with Alport syndrome who can develop anti-GBM disease following a renal transplant. Other laboratory tests include antineutrophil cytoplasmic antibodies (ANCA) levels which are positive in 15% to 30% of the cases. Amongst this proportion, 75% of the cases are perinuclear anti-neutrophil cytoplasmic antibodies (P-ANCA) positive while the remaining are positive for cytoplasmic antineutrophil cytoplasmic antibodies (C-ANCA). Our patient was found to be positive for all said markers of the disease. While these non-invasive tests lead to much insight about the underlying pathology, a renal biopsy remains the gold standard for establishing a diagnosis. A biopsy is recommended, even if the anti-GBM antibodies are positive, in order to assess the severity of fibrosis at the time of the presentation [5,7-8].

Kidney biopsy samples from a large biopsy series revealed that 95% of the patients with anti-GBM disease demonstrated a crescent formation and more than 50% of the glomeruli were involved in 80% of the patients. The hypothesis is that the number of crescents in a biopsy correlates with the severity of renal impairment at the time of presentation [4]. A microscopic analysis in our patient revealed granulomatous crescentic glomerulonephritis (Figure 3), and subsequent immunofluorescence of the histological section showed pathognomonic linear deposits of IgG along the basement membrane (Figure 4).

Treatment of anti-GBM disease includes plasmapheresis which can be complemented with corticosteroids and cyclophosphamide to achieve the maximal efficacy of the therapy. Cui et al. conducted a study on 221 patients with anti-GBM disease and described better outcomes in patients who were treated with a combination of plasmapheresis, cyclophosphamide, and pulse therapy with corticosteroids. Addition of high dose prednisone (1 mg/kg/day) which is gradually tapered over the course of the next twelve months allows for further suppression of the antibody-mediated glomerulonephritis. Anti-GBM antibodies should be monitored and plasmapheresis can be discontinued once the auto-antibody levels become undetectable [9].

Despite effective treatment modalities, the survival rates vary proportionately with the severity of the renal injury. A retrospective review of patients treated for a confirmed anti-GBM disease for over 25 years concluded that serum creatinine was one of the most essential predictors of survival with a five-year patient and renal survival rate as high as 94% if serum creatinine levels are < 5.65 mg/dL. The five-year renal survival dropped to 50% in patients with higher values. The one-year survival was dismayingly as low as 1% in patients who required hemodialysis at the time of their initial presentation. Some of the other predictors of poor renal outcome included a high proportion of crescentic glomeruli, severe renal dysfunction, and oliguria at the time of presentation [10]. 

## Conclusions

Isolated renal involvement in anti-glomerular basement membrane disease is a rare clinical entity. The patient may present with fluctuating levels of renal impairment, but acute renal failure secondary to crescentic glomerulonephritis is the typical exhibition of the disease process. The hastened clinical deterioration should raise suspicion in treating physicians who should promptly initiate management with a combination of plasmapheresis, cyclophosphamide, and corticosteroids to ensure optimal recuperation. 
